# Recurrence of Disease Following Liver Transplantation: Nonalcoholic Steatohepatitis vs Hepatitis C Virus Infection

**Published:** 2011-05-01

**Authors:** I. A. Hanouneh, C. Macaron, R. Lopez, A. E. Feldstein, L. Yerian, B. Eghtesad, N. N. Zein

**Affiliations:** 1 *Department of Gastroenterology and Hepatology, *; 2 *Department of Quantitative Health Sciences, *; 3 *Department of Anatomic Pathology, *; 4 *Department of General Surgery, Transplant Center, The Cleveland Clinic, Cleveland, Ohio, USA*

**Keywords:** Nonalcoholic steatohepatitis, Hepatitis C virus, Orthotopic liver transplantation, Prognosis

## Abstract

Background: Nonalcoholic steatohepatitis (NASH) is an increasing indication for orthotopic liver transplantation (OLT) in the United States and other countries. However, the incidence of disease recurrence and natural course following OLT remains incompletely understood.

Objective: To estimate the incidence of recurrent disease, outcome and identify risk factors associated with disease recurrence in patients undergoing OLT for NASH as compared to those undergoing OLT for HCV cirrhosis.

Methods: We identified all patients with end-stage liver disease secondary to NASH (n=53) or HCV (n=95) cirrhosis who underwent OLT at our institution between 1998 and 2005. Protocol liver biopsies were performed (Day 7, Month 4 and yearly) after OLT, and as clinically indicated. Kaplan-Meier survival analysis was performed to assess the fibrosis progression and survival. Cox regression analysis was performed to identify factors associated with disease recurrence.

Results: Five-year survival was 90.5% in NASH vs 88.4% in HCV group (p=0.97). The median (25%ile, 75%ile) follow-up to last available biopsy was 12.7 (5.9, 26.3) months, during which 17 (32%) of NASH patients developed persistent fatty infiltration in their graft, 8 (15%) of whom had accompanying histologic features of recurrent NASH. There was no difference in the prevalence of post-OLT steatosis between HCV and NASH patients after adjusting for time of histologic follow-up (p=0.33). Patients with HCV infection were more likely to develop hepatic fibrosis post-OLT than those with NASH (62.1% vs 18.9%, p<0.001). Multivariate analysis identified post-OLT diabetes (HR=2.0, 95% CI: 1.2–3.2, p=0.007) as an independent risk factor for fibrosis development. Additionally, NASH subjects who received steroids had a significantly higher risk of developing hepatic fibrosis post-OLT than NASH patients who did not receive steroids and all HCV subjects (p<0.001).

Conclusion: Recurrence of steatosis post-OLT is common. Corticosteroid use may contribute to fibrosis progression in this population.

## INTRODUCTION

Nonalcoholic fatty liver disease (NAFLD) and nonalcoholic steatohepatitis (NASH) have emerged as common causes of chronic liver disease worldwide. It is estimated that by the year 2025, more than 25 million Americans may have NASH-related chronic liver disease [1]. This prevalence would greatly exceed the current prevalence of hepatitis C virus (HCV)-related liver disease [1]. NASH can progress to cirrhosis and liver failure necessitating orthotopic liver transplantation (OLT) in 3%–15% of patients [2, 3]. Given these current estimates, we would predict increased demand for OLT due to NASH cirrhosis. However, little is known about the frequency of disease recurrence and eventual outcome of the liver allograft in patients with NASH cirrhosis undergoing OLT. The identification of the natural course of NASH and modifiable risk factors that promote disease recurrence post-OLT is therefore of paramount importance for any strategy aimed at improving outcomes.

The recurrence of HCV infection post-OLT is a universal event [4, 5], whereas the natural course of NASH post-OLT is less clearly identified. Small series have demonstrated 11%–38% recurrence rate of NASH post-OLT [6-9]. Several recipient factors influence the recurrence of NASH post-OLT. The metabolic syndrome (MS), characterized by a constellation of factors including obesity, diabetes mellitus, hypertension, and hyperlipidemia, is the most frequent risk factor for the development of NAFLD and NASH. These metabolic derangements often occur after OLT and have become well recognized side effects of immunosuppressant agents used in OLT. In one series of 95 patients with HCV who underwent OLT [10], there was a significant increase in the prevalence of metabolic derangements including hypertension and diabetes mellitus post-OLT compared to pre-transplant characteristics.

Other factors that may influence the recurrence of NAFLD and NASH post-OLT are immunosuppressant agents, specifically corticosteroids. In fact, corticosteroids are frequently implicated in the pathogenesis of NAFLD and NASH [11, 12]. In one small series [12], cumulative steroid use has been associated with recurrent NASH post-OLT. The objectives of this study are to estimate the incidence of recurrent disease, patient and liver allograft outcome, and identify risk factors associated with recurrent disease and fibrosis progression in patients undergoing OLT for NASH compared to those undergoing OLT for HCV.

## PATIENTS AND METHODS

Using our electronic pathology database, we reviewed the medical records of all adult patients aged >18 years who underwent OLT for end-stage liver disease secondary to NASH or HCV cirrhosis at the Cleveland Clinic between 1998 and 2005. The presence of HCV was confirmed by qualitative seropositivity for HCV RNA. NASH cirrhosis was defined as histologic features consistent with NASH on liver explants, or cryptogenic cirrhosis in the setting of MS. All other causes of liver disease were ruled out as part of the routine pre-OLT evaluation. 

MS was defined at one year post-OLT according to the National Cholesterol Education Program, Adult Treatment Panel III (ATP III) [13]. The “waist circumference” trait in the ATP III criteria was replaced by body mass index (BMI) greater than 28.8 kg/m^2^ in both men and women for the purpose of this study. This cutoff value for BMI was equivalent in a regression analysis to a waist circumference of 102 cm in cross-sectional studies of men in the United States and in Europe [14].

Recipients of living donor grafts were excluded. None of our patients received donor livers with greater than 30% steatosis. We also excluded subjects who have been referred for a second OLT or multi-solid organ transplants during the time period from which study patients were identified. 

Demographic data (patient’s age, gender and race), donor age, stage of post-transplant liver biopsies, biopsy-proven acute cellular rejection (ACR) and corresponding treatment were extracted from prospectively collected data for patients with NASH or HCV who underwent OLT at the Cleveland Clinic. Additionally, details of metabolic traits including BMI, fasting serum glucose, triglyceride and HDL, systolic and diastolic blood pressure at one year after OLT were recorded. Detailed medical history of hypertension, hyperlipidemia, and diabetes, and their corresponding therapy were also obtained from medical records.

Histological analysis:

Protocol liver biopsies were performed (day 7, month 4 and yearly) after OLT. Additional biopsies were performed as clinically indicated. All biopsies were read by dedicated liver pathologists. The Banff schema [15] was used to evaluate ACR, and accordingly defined as moderate or severe ACR when the rejection activity index score was ≥5. Hepatic fibrosis (stage) was assessed by Ludwig-Batts scoring system [16], and accordingly assigned using a scale of 0 to 4 (F0: absent, F1: portal fibrosis, F2: periportal fibrosis, F3: bridging fibrosis, F4: cirrhosis). The first biopsy that showed at least stage one fibrosis post-OLT was used for the time-to-progression analysis assuming F0 for all patients at the time of OLT. If there was no evidence of fibrosis during post-OLT follow-up, the last biopsy performed was chosen for the time-to-progression analysis. 

Hepatic steatosis was quantified as the percentage of the hepatic parenchyma occupied by fat droplets, and accordingly assigned as negative when <5% of the hepatic parenchyma was involved, and positive when there was ≥5% involvement. The presence of steatosis in combination with two of the following features accounts for the diagnosis of NASH. These histological features include, lobular inflammatory cell infiltrates, ballooning hepatocyte degeneration, Mallory’s hyaline, and a typical pattern of fibrosis.

Immunosuppression:

Induction therapy at the Cleveland Clinic consists of two doses of interleukin-2 receptor antagonist (Basiliximab).The first dose (20 mg) is administered in the intensive care unit, within 12 hours of transplantation. Patients receive the second dose on postoperative day 4 (20 mg). All patients receive 1000 mg intravenous methylprednisolone in the operating room followed by a tapering dose of intravenous methylprednisolone to 20 mg/day by day 6. This is followed by conventional steroid taper of oral prednisone until discontinued by day 21 following transplantation. 

Calcineurin inhibitors are administered to all patients. Mycophenolate mofetil is added when side effects precluded full therapeutic dose of calcineurin inhibitors.

Mild ACR is treated by increasing tacrolimus trough level, while corticosteroids are reserved for those with moderate or severe rejection (rejection activity index ≥5). Patients with biopsy-proven moderate or severe ACR are treated with an intravenous bolus of 1000 mg methylprednisolone followed by tapering dose of intravenous methylprednisolone or oral prednisone over five days. 

Intravenous valganciclovir is administered for a minimum of seven days post-transplantation as prophylaxis against cytomegalovirus (CMV) infection. No CMV prophylaxis is given to the sero-negative donor/sero-negative recipient (D-/R-) combination.

Statistical Analysis:

Descriptive statistics were computed for all factors. These include means, standard deviations and percentiles for continuous variables and frequencies for categorical factors. Univariate analysis was done to compare subjects with NASH to those with HCV. Wilcoxon rank sum tests were used to compare continuous and Pearson’s χ^2^ were used for categorical variables. The same was done to compare subjects who developed steatosis during follow-up to those who did not. In addition, a binary logistic regression analysis was performed to assess factors associated with disease recurrence post-OLT. Subject group and duration of follow-up were forced in the models and an automated stepwise variable selection was performed on 1000 bootstrap samples to choose the final model; factors that appeared in ≥30% of replications were kept in the final model. Interactions between disease group and all the variables in the final model were explored and included if p<0.1. 

A time-to-event analysis was performed to assess differences in fibrosis progression and survival between the two groups. Time of follow-up was defined as number of years from OLT to either occurrence of event or last follow-up visit if no event. Kaplan-Meier survival analysis and log-rank tests were used to compare the two groups. For determing time-to-progression, univariate and multivariate Cox proportional analysis were also performed. The same variable selection method as described above was utilized to build the final model. 

A p<0.05 was considered statistically significant. SAS ver 9.2 software (The SAS Institute, Cary, NC) and R ver 2.4.1 software (The R Foundation for Statistical Computing, Vienna, Austria) were used to perform all analyses.

## RESULTS

Baseline characteristics:

A total of 148 patients who had liver transplantation for NASH cirrhosis (n=53) or HCV cirrhosis (n=95) during the study period were identified. The baseline characteristics of our study population are listed in Table 1. The median (25%ile, 75%ile) age of the recipient at the time of the transplant for the entire study population was 55.4 (50.0, 60.0) years; the studied patients consisted of 112 (75.7%) men, and 119 (80.4%) Caucasians. Patients with NASH were older than those with HCV at the time of the transplant (57.8 *vs* 54.0 years, p=0.005). Significantly fewer NASH patients were males: (32 (60%) NASH compared to 80 (84%) HCV patients, p=0.001). Additionally, the HCV group was composed of fewer Caucasians than the NASH group (73% *vs* 93%, p=0.006).

**Table 1:** Demographic and clinical characteristics by disease group

**Factor**	**All(n=148)**	**NASH(n=53)**	**HCV(n=95)**	**p value**
Male	112 (75.7)	32 (60.4)	80 (84.2)	0.001
Caucasian	119 (80.4)	49 (92.5)	70 (73.7)	0.006
Age	55.4 (50.0, 60.0)	57.8 (52.3, 63.4)	54.0 (50.0, 58.0)	0.005
Triglyceride (n=98)	153.5 (106.0, 208.0)	150.0 (115.0, 200.5)	153.5 (104.0, 212.0)	0.99
HDL (n=96)	43.0 (35.0, 52.0)	43.0 (37.5, 51.0)	43.0 (34.0, 54.5)	0.91
Tacrolimus	128 (86.5)	48 (90.6)	80 (84.2)	0.28
Cyclosporine	13 (8.8)	4 (7.6)	9 (9.5)	0.77
Mycophenolate mofetil	53 (35.8)	21 (39.6)	32 (33.7)	0.47
Diabetes post-OLT	87 (58.8)	38 (71.7)	49 (51.6)	0.017
Hypertension post-OLT	89 (60.1)	28 (52.8)	61 (64.2)	0.18
BMI @1 yr post-OLT (n=136)	28.6 (24.3, 31.9)	30.9 (26.7, 34.1)	27.1 (23.9, 30.1)	<0.001
MS @1 yr post-OLT (n=123)	65 (52.9)	24 (58.5)	41 (50.0)	0.37
Acute cellular rejection	65 (43.9)	22 (41.5)	43 (45.3)	0.66
Steroids	34 (23.6)	3 (5.7)	31 (34.1)	<0.001
Total FU months (OLT to last Bx)	12.7 (5.9, 26.3)	5.1 (0.69, 13.4)	19.6 (11.9, 30.0)	<0.001
Steatosis	64 (43.2)	17 (32.1)	47 (49.5)	0.041
Fibrosis	69 (46.6)	10 (18.9)	59 (62.1)	<0.001

Values are presented as n(%) or Median (25%ile, 75%ile)

p values correspond to Pearson’s ϰ^2^ tests for categorical factors and Wilcoxon rank sum tests for continuous variables

All patients received deceased donor liver allograft with a median (25%ile, 75%ile) donor age of 46.0 (34.0, 62.0) year. Immunosuppressive therapy was primarily tacrolimus in 128 (86.5%) patients, while 13 (8.8%) subjects received cyclosporine-based regimen. The two study groups (NASH and HCV) were comparable with regard to the choice of calcineurin inhibitor. Mycophenolate mofetil (MMF) was used as an adjuvant medication in 53 (35.8%) subjects. Of the entire study population, biopsy-proven ACR occurred in 65 (43.9%) patients, of whom 34 (23.6%) required intravenous steroid therapy. The rate of ACR was not significantly different between NASH and HCV patients (41.5% *vs* 45.3%, p=0.66). However, only three (6%) patients in the NASH group developed moderate to severe ACR that required intravenous steroid therapy, compared to 31 (34%) of HCV patients (p<0.001).

The prevalence of metabolic features post-OLT:

MS, defined at one year post-OLT, was present in 65 (52.9%) of our entire study population. Compared to pre-OLT characteristics, we observed a significant increase in the frequency of diabetes (46% pre-OLT *vs* 58.8% post-OLT, p=0.006) and hypertension (49% pre-OLT *vs* 60.1% post-OLT, p=0.004) after OLT.

The prevalence of diabetes mellitus, defined at one year post-OLT, was more common in NASH compared to HCV patients (38 (71.7%) *vs* 49 (51.6%), p=0.017). Additionally, the median (25%ile, 75%ile) BMI at one year post-OLT was greater in NASH (30.9 (26.7, 34.1) kg/m^2^) than HCV patients (27.1 (23.9, 30.1) kg/m^2^) (p<0.001). The rate of hypertension and hyperlipidemia was not different between the two groups. As such, there was no significant difference in the prevalence of MS at one year post-OLT between the NASH and HCV patients, (58% *vs* 50%, p=0.37). Furthermore, there was no association between the development of post-OLT MS and gender, ethnicity, or type of immunosuppressive treatment regimen.

Recurrence of hepatic steatosis post-OLT:

Histologic follow-up was available in all subjects who underwent OLT for NASH cirrhosis with a total of 159 post-OLT liver biopsies performed. The median (25%ile, 75%ile) time of histologic follow-up to last available biopsy post-OLT was 5.1 (0.69, 13.4) months, during which 17 (32%) of NASH patients developed persistent fatty infiltration in their graft, 8 (15%) of whom had accompanying histologic features consistent with recurrent NASH.

The HCV population (n=95) was histologically followed over a median (25%ile, 75%ile) time of 19.6 (11.9, 30.0) months, during which 408 post-OLT liver biopsies performed. In fact, there was no significant difference in the prevalence of post-OLT steatosis between HCV and NASH patients after adjusting for time of histologic follow-up (p=0.33). 

Table 2A shows the factors associated with development of post-OLT steatosis in univariate analysis. The risk of developing hepatic steatosis post-OLT significantly increased with time (p<0.001). Other factors including age, gender, metabolic features, type of calcineurin inhibitor, and the use of steroids for ACR were not significantly associated with the development of steatosis. In multivariate Cox regression analysis (Table 2B), longer follow-up time was the only factor independently associated with the development of steatosis post-OLT (p=0.004). Due to the small number of patients who developed recurrent NASH post-OLT (n=8), we were not been able to study the factors associated with NASH post-OLT. 

**Table 2A:** Factors associated with steatosis—univariate analysis

**Factor**	**Steatosis (n=64)**	**No Steatosis (n=84)**	**Duration of FU-Unadjusted p value**	**Duration of FU-Adjusted p value**
Underlying diagnosis (HCV *vs* NASH)	17 (26.6)	36 (42.9)	***0.041***	0.33
Male	47 (73.4)	65 (77.4)	0.58	0.32
Caucasian	52 (81.3)	67 (79.8)	0.82	0.65
Age	55.0 (50.7, 59.0)	56.0 (50.0, 63.0)	0.38	0.34
Triglyceride	157.0 (115.0, 219.0)	153.0 (101.0, 206.0)	0.65	0.56
HDL	42.0 (34.0, 50.0)	46.0 (39.0, 53.0)	0.14	0.19
Tacrolimus	56 (87.5)	72 (85.7)	0.75	0.56
Cyclosporine	5 (7.8)	8 (9.5)	0.72	0.41
Mycophenolate mofetil	25 (39.1)	28 (33.3)	0.47	0.12
Diabetes post-OLT	39 (60.9)	48 (57.1)	0.64	0.21
Hypertension post-OLT	37 (57.8)	52 (61.9)	0.61	0.65
BMI @1 yr post-OLT	28.4 (25.0, 31.6)	28.7 (24.0, 32.1)	0.97	0.97
MS @1 yr post-OLT	31 (53.5)	34 (52.3)	0.9	0.66
Acute cellular rejection	26 (40.6)	39 (46.4)	0.48	0.16
Steroids	17 (27.4)	17 (20.7)	0.35	0.99


Values are presented as n(%) or median (25%ile, 75%ile)

Unadjusted p values correspond to Pearson’s χ^2^ tests for categorical factors and Wilcoxon rank sum tests for continuous variables. Adjusted p values correspond to logistic regression analysis adjusting for time between OLT and last biopsy.

**Table 2B:** Factors associated with steatosis—multivariate analysis

**Factor**	**OR (95% CI)**	**p value**
HCV *vs* NASH	1.7 (0.75–3.7)	0.20
Years to last FU	1.5 (1.1–1.9)	0.004
Female	1.7 (0.75–3.9)	0.20

**Figure 1:**
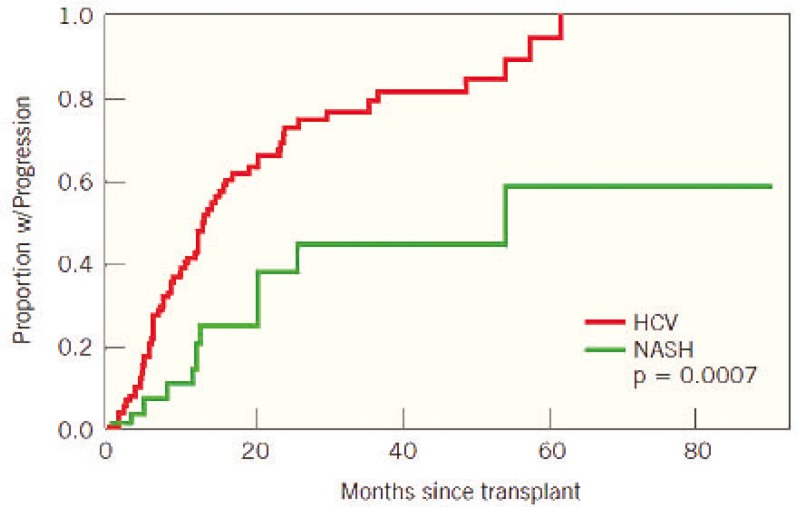
Fibrosis progression post-OLT in patients with HCV and NASH

Recurrence of hepatic fibrosis post-OLT:

Of 148 patients, 69 (46.6%) developed fibrosis post-OLT, while 79 (53.4%) remained fibrosis free post-OLT. Patients with HCV were more likely to develop hepatic fibrosis post-OLT than those with NASH, 62.1% *vs* 18.9% (p<0.001). The median (25%ile, 75%ile) rate of fibrosis progression in the HCV and NASH was 0.5 (0.0, 1.3) and 0.0 (0.0, 0.0) fibrosis stage per year, respectively (p<0.001) (Figure 1). Furthermore, 19 (20%) HCV patients developed advanced hepatic fibrosis (stage 3 or 4) post-OLT, compared to only one patient (2%) in the NASH group (p=0.002). After adjusting for length of histologic follow-up, HCV patients remained at higher risk of developing fibrosis post-OLT compared to those with NASH (p=0.0007). Table 3A shows the factors associated with fibrosis progression in univariate analysis. The hazard for fibrosis progression post-OLT was higher in subjects with HCV than those with NASH (HR=2.1; 95% CI: 1.1–3.9). Additionally, the use of steroids for ACR was associated with a higher hazard for fibrosis progression (HR=1.9, p=0.009). Other factors associated with fibrosis progression post-OLT were hypertension (p=0.044), diabetes mellitus (p=0.008), and MS (p=0.019). 

**Table 3A:** Factors associated with fibrosis—univariable analysis

**Factor**	**Progression(n=79)**	**No Progression(n=69)**	**Hazard Ratio(95% CI)**	**p value**
Underlying diagnosis (HCV *vs* NASH)	68 (86.1)	27 (39.1)	2.1 (1.1, 3.9)	***0.025***
Male	67 (84.8)	45 (65.2)	1.6 (0.86, 3.0)	0.14
Non-Caucasian	22 (27.9)	7 (10.1)	1.6 (0.95, 2.6)	0.078
Age	55.0 (49.0, 58.0)	57.0 (52.0, 62.3)	0.98 (0.95, 1.02)	0.31
Triglyceride	137.0 (92.0, 200.0)	174.0 (125.0, 224.0)	0.998 (0.994, 1.001)	0.11
HDL	43.0 (35.0, 55.0)	43.0 (35.0, 51.0)	1.01 (0.99, 1.02)	0.21
Tacrolimus	68 (86.1)	60 (87.0)	0.95 (0.50, 1.8)	0.87
Cyclosporine	8 (10.1)	5 (7.3)	1.1 (0.54, 2.4)	0.73
Mycophenolate mofetil	25 (31.7)	28 (40.6)	1.1 (0.70, 1.8)	0.6
Diabetes post-OLT	49 (62.0)	38 (55.1)	1.9 (1.2, 3.0)	***0.008***
Hypertension post-OLT	51 (64.6)	38 (55.1)	1.6 (1.01, 2.6)	***0.044***
BMI @1 yr post-OLT	27.6 (24.0, 31.2)	29.0 (25.1, 32.9)	0.96 (0.92, 1.01)	0.12
MS @1 yr post-OLT	40 (59.7)	25 (44.6)	1.8 (1.1, 3.0)	***0.019***
Acute cellular rejection	39 (49.4)	26 (37.7)	1.3 (0.84, 2.0)	0.24
Steroids	28 (37.3)	6 (8.7)	1.9 (1.2, 3.0)	***0.009***

Values are presented as n(%) or median (25%ile, 75%ile)

**Figure 2:**
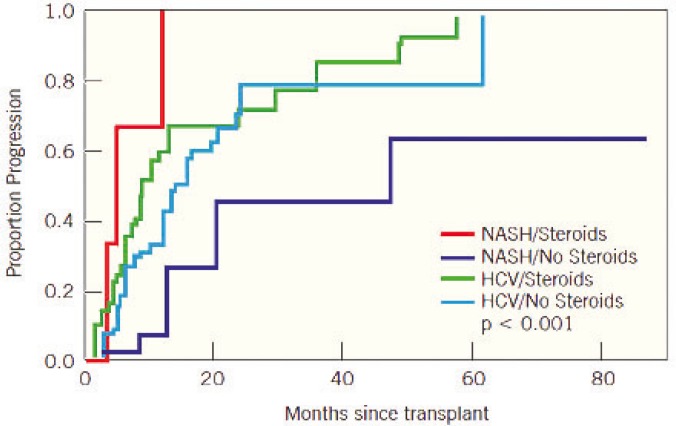
Fibrosis progression post-OLT after adjusting for steroid use and disease group

In multivariate analysis, after adjusting for steroid use and disease group, subjects with diabetes had twice the hazard of developing fibrosis than those without diabetes (p=0.007). As stated previously, NASH patients were at lower risk of developing fibrosis post-OLT than HCV patients. Interestingly, multivariate analysis showed that the use of corticosteroids in NASH patients carries a risk of developing fibrosis post-OLT that rivals the risk in patients with recurrent HCV post-OLT (Table 3B). NASH subjects who received steroids for the treatment of ACR had a significantly higher hazard of developing hepatic fibrosis post-OLT than NASH patients who did not receive steroids. The interaction between post-OLT liver fibrosis and the steroid use for ACR was not statistically significant in the HCV population (Figure 2).

**Table 3B:** Factors associated with fibrosis—multivariable analysis

**Factor**	**Hazard Ratio(95% CI)**	**p value**
HCV/No Steroids *vs* NASH/No Steroids	2.5 (1.2–5.5)	0.018
HCV/Steroids. NASH/No Steroids	3.2 (1.4–7.2)	0.005
NASH/Steroids *vs* NASH/No Steroids	8.1 (2.1–31.5)	0.003
Diabetes	2.0 (1.2–3.2)	0.007
Non-Caucasian	1.4 (0.82–2.3)	0.23

Survival outcome post-OLT:

At a median (25%ile, 75%ile) follow-up of 55.4 (43.6, 60) months post-OLT, the overall survival in the entire study population was 90% (Figure 2). To estimate the impact of recurrent disease on survival outcome post-OLT, we excluded deaths that occurred in the perioperative period (within six months of OLT). As such, five-year survival was not significantly different between NASH and HCV patients (90.5% *vs* 88.4%, p=0.97). Causes of death in patients who underwent transplant for NASH cirrhosis were acute lymphoblastic lymphoma (n=1), melanoma (n=1), pneumonia (n=1), mesenteric ischemia (n=1) and unknown (n=1). None of the NASH patients died of complications related to end-stage liver disease post-OLT. In contrast, 11 (12%) of HCV patients died, eight of whom (8%) due to complications related to end-stage liver disease. Two other patients with HCV post-OLT died of recurrent hepatocellular carcinoma. The cause of death was unknown in one subject. Accordingly, portal hypertension and cirrhosis was the most common cause of death post-OLT in the HCV population and significantly more common in the HCV compared to NASH patients (8/11 (8%) *vs* 0/5 (0%), p<0.001).

## DISCUSSION

NAFLD is emerging as an important cause of cirrhosis and hepatocellular carcinoma accounting for a significant proportion of OLTs performed in the United States [1-3]. Little is known about the natural history of NASH post-OLT. Over a median (25%ile, 75%ile) histological follow-up period of 5.1 (0.69, 13.4) months, our study reported the rate of recurrent NASH post-OLT of 15.1% in patients with cryptogenic cirrhosis who had clinical or histological features suggestive of NASH. Although the majority of patients who experienced recurrent NASH post-OLT have benign course, a small number of patients may have progressive disease leading to cirrhosis and require second OLT. In contrast, recurrence of HCV post-OLT is a universal event that frequently leads to serious consequences including cirrhosis and liver failure and has emerged as an important cause of retransplantation in the United States [4, 5].

Among the many factors that may potentially predispose to the recurrence of NASH post-OLT [7, 8], immunosuppression is of particular interest. Corticosteroids are frequently implicated in the pathogenesis of steatosis and steatohepatitis [11, 12]. It has been demonstrated that cumulative use of steroids is associated with recurrent NASH post-OLT [12]. A strong correlation between the use of corticosteroids for ACR and the development of fibrosis post-OLT in patients undergoing liver transplant for NASH cirrhosis was well documented in our study. Our study suggests that the use of corticosteroids post-OLT in NASH carries a risk of developing hepatic fibrosis that rivals the risk in patients with post-OLT recurrent HCV. Based on these findings, immunosuppressant protocols for NASH patients undergoing OLT should minimize the use of corticosteroids when possible. The choice of calcineurin inhibitor (tacrolimus *vs* cyclosporine) and the use of MMF were not associated with progression of fibrosis post-OLT in NASH patients. 

Diabetes mellitus, hypertension, hyperlipidemia and obesity, which are risk factors of NASH, are common after OLT [10]. In our series, we observed a significant increase in the frequency of hypertension and diabetes after OLT. These metabolic factors are known to contribute to NASH. Due to the small number of patients who experienced recurrent NASH post-OLT, we were not been able to study the factors associated with recurrent NASH. However, our study identified a statistically significant association between diabetes mellitus, hypertension and MS and the development of fibrosis post-OLT in both HCV and NASH patient groups. 

It has previously been shown that steatosis associated with HCV genotype 1 infection is a marker of metabolic abnormalities including obesity, hyperlipidemia or diabetes mellitus and has been termed “metabolic fat” [17- 20]. In contrast, steatosis in patients with HCV genotype 3 is associated with viremia rather than metabolic abnormalities and has been termed “viral fat” [17-20]. Our study showed no significant difference in the prevalence of steatosis in patients with HCV or NASH after adjusting for the time of histological follow-up post-OLT. This finding still remained true after excluding patients with HCV genotype 3 (n=10). Furthermore, this relationship between HCV and steatosis was independent of the metabolic derangements as the latter were not associated with the development of steatosis in our series. It is possible that the development of steatosis is virologically mediated. As such, our findings support emerging data that HCV promotes the development of insulin resistance and hepatic steatosis [17-20].

In conclusion, recurrence of hepatic steatosis and fibrosis post-OLT is common. Corticosteroid use may contribute to fibrosis progression in this population.
